# Hepatic Steatosis in Pulsed Field Ablation for Atrial Fibrillation: Prevalence, Liver Marker Concordance and Acute Procedural Characteristics

**DOI:** 10.3390/jcm15176805

**Published:** 2026-09-02

**Authors:** Maria Boszko, Bartosz Krzowski, Michał Gawlik, Alicja Skrobucha, Julia Szydlik, Mateusz Wawrzeńczyk, Karolina Mitrzak, Kacper Rutkowski, Karolina Ignaczak, Monika Gawałko, Cezary Maciejewski, Jakub Rokicki, Michał Peller, Michał Marchel, Piotr Lodziński, Grzegorz Styczyński, Marcin Grabowski, Paweł Balsam

**Affiliations:** 1 1st Chair and Department of Cardiology, Medical University of Warsaw, 02-097 Warsaw, Poland; maria.boszko@wum.edu.pl (M.B.);; 2 Department of Cardiology, Cardiovascular Research Institute Maastricht, Maastricht University Medical Centre, 6200 MA Maastricht, The Netherlands; 3 Department of Medical Informatics and Telemedicine, Medical University of Warsaw, 02-097 Warsaw, Poland; 4 Department of Internal Medicine, Hypertension and Vascular Diseases, Medical University of Warsaw, 02-097 Warsaw, Poland

**Keywords:** atrial fibrillation, pulsed field ablation, catheter ablation, hepatic steatosis, metabolic dysfunction-associated steatotic liver disease, hepato-renal index

## Abstract

**Background/Objectives:** Hepatic steatosis and metabolic dysfunction-associated steatotic liver disease (MASLD) are cardiometabolic phenotypes relevant to atrial fibrillation (AF), but their prevalence and acute procedural relevance in pulsed field ablation (PFA) are poorly defined. We estimated the prevalence of hepato-renal index (HRI)-detected hepatic steatosis, assessed liver marker concordance, and explored acute procedural characteristics. **Methods:** In this single-centre prospective study, 99 consecutive eligible patients undergoing first-time PFA for AF underwent B-mode hepatic ultrasound. Hepatic steatosis was defined a priori as HRI ≥ 1.28, measured offline by an investigator blinded to clinical and procedural data. HRI-based MASLD-compatible phenotype, Hepatic Steatosis Index (HSI), BARD score, and Fibrosis-4 index were analysed as secondary liver markers. **Results:** HRI-detected hepatic steatosis was present in 62/99 patients (62.6%, 95% CI 52.8–71.5), and HRI-based MASLD-compatible phenotype in 55/99 (55.6%, 95% CI 45.7–65.0). Agreement between HRI and HSI was poor (Cohen’s κ = 0.01; prevalence-adjusted bias-adjusted κ +0.06; positive agreement 62%). Acute pulmonary vein isolation was achieved in all patients. First-pass isolation was high in both groups (91.9% vs. 81.1%; *p* = 0.124). Most procedural metrics were similar. Fluoroscopy dose was modestly higher in patients with steatosis (median 226 vs. 207 mGy; *p* = 0.035), but this exploratory signal was inconsistent across sensitivity analyses. In-hospital complications were infrequent (4/99, 4.0%). **Conclusions:** In first-time PFA patients with feasible HRI assessment, hepatic steatosis was common, liver marker classifications showed limited patient-level agreement, and acute procedural conduct showed no consistent steatosis-specific signal. Long-term rhythm durability requires dedicated follow-up.

## 1. Introduction

Atrial fibrillation (AF) is the most common sustained arrhythmia in adults and a major contributor to cardiovascular morbidity and mortality worldwide [1]. Pulsed field ablation (PFA) has emerged as a non-thermal, tissue-selective alternative to conventional thermal ablation, with randomised and registry data supporting high acute efficacy and a favourable safety profile [2,3]. The 2026 EHRA/HRS/APHRS/LAHRS/CHRS Scientific Statement on PFA summarises the current evidence base for PFA and emphasises that differential benefit or risk across specific patient subgroups remains insufficiently characterised [4]. Hepatic steatosis and metabolic dysfunction-associated steatotic liver disease (MASLD) represent common cardiometabolic phenotypes in AF, but their prevalence and relationship with acute procedural conduct during PFA remain poorly defined.

MASLD, redefined under the 2023 multisociety Delphi consensus, is among the most prevalent chronic liver conditions worldwide and is increasingly recognised as a systemic metabolic disorder with cardiovascular consequences [5,6]. In patients undergoing AF ablation, hepatic steatosis may serve as a marker of a broader metabolic phenotype, including greater body habitus, insulin resistance, low-grade inflammation, and clustering of cardiovascular risk factors. These features could plausibly affect acute procedural workflow, radiation exposure, or periprocedural vulnerability, without necessarily implying a direct effect of steatosis on PFA lesion formation. In AF populations, MASLD has been associated with adverse cardiovascular outcomes, supporting its relevance as a clinically meaningful cardiometabolic phenotype rather than an incidental hepatic finding [7].

Evidence linking hepatic steatosis or MASLD to AF ablation has so far focused mainly on thermal ablation and long-term arrhythmia recurrence, whereas acute procedural characteristics and periprocedural safety have not been the primary focus [8,9]. Whether these findings translate to the acute procedural context of PFA remains unknown. We therefore evaluated, in a prospective cohort of patients undergoing first-time PFA for AF, the prevalence of HRI-detected hepatic steatosis as the primary descriptive objective. Agreement between non-invasive liver markers was assessed as a secondary objective, while acute procedural characteristics and in-hospital complications were examined exploratorily. Long-term rhythm outcomes and arrhythmia-free survival were considered a distinct prognostic question and were not part of the present acute procedural analysis.

## 2. Materials and Methods

### 2.1. Study Design and Population

This single-centre prospective observational study was conducted between December 2023 and March 2025 at the 1st Chair and Department of Cardiology, Medical University of Warsaw, a tertiary cardiac centre. Consecutive eligible patients with paroxysmal or persistent AF referred for first-time PFA were enrolled. Patients were excluded if they had undergone prior left atrial ablation, had known chronic liver disease, or did not have reliable HRI assessment. Known chronic liver disease was identified from medical records and included previously documented chronic hepatic conditions (e.g., cirrhosis, hepatitis). Unreliable HRI assessment was defined as estimated glomerular filtration rate <60 mL/min/1.73 m^2^, absence of the right kidney, or technically inadequate ultrasound imaging precluding standardised liver–kidney echogenicity measurement. The renal criterion was applied because the renal cortex serves as the internal echogenicity reference for HRI, and altered renal cortical echogenicity in chronic kidney disease may systematically bias the liver-to-kidney ratio.

### 2.2. Assessment of Hepatic Steatosis and HRI-Based MASLD-Compatible Phenotype

On admission, hepatic steatosis was assessed using the hepato-renal index (HRI) derived from B-mode ultrasound images of the right liver lobe and adjacent right renal cortex. Images were acquired from a right lateral approach in the supine position and analysed offline using ImageJ software (version 1.54p National Institutes of Health, Bethesda, MD, USA). HRI was calculated as the ratio of mean liver to renal cortical echogenicity measured within standardised, depth-matched regions of interest by an investigator blinded to clinical, procedural and outcome data. Hepatic steatosis was defined a priori as HRI ≥ 1.28, a threshold derived from a computer-assisted sonographic validation study against histology [10]. Because published HRI thresholds vary across validation cohorts, reference standards, and ultrasound acquisition protocols, the primary HRI ≥ 1.28 threshold was interpreted as a sensitive, screening-oriented imaging threshold rather than a definitive diagnostic cut-off. Sensitivity analyses using more conservative HRI thresholds reported in the literature were performed; details of threshold selection are provided in the Supplementary Methods and Table S3. Detailed image acquisition, region-of-interest placement, quality control procedures and intraobserver reproducibility assessment are provided in the Supplementary Methods.

HRI-based MASLD-compatible phenotype was analysed as a pragmatic secondary clinical classification and was defined as HRI-detected hepatic steatosis plus at least one cardiometabolic risk criterion available in the study database, in line with the 2023 multisociety MASLD framework [5]. Cardiometabolic criteria included overweight/obesity or increased waist circumference, dysglycaemia or type 2 diabetes, elevated blood pressure or treated hypertension, hypertriglyceridemia or lipid-lowering therapy, and low HDL cholesterol, where available. This classification was intended to identify a liver metabolic study phenotype compatible with MASLD, rather than to provide exhaustive hepatological phenotyping or full steatotic liver disease subclassification. The Hepatic Steatosis Index (HSI; ≥36) was calculated as a secondary steatosis-related index [11]. The BARD score (≥3) and Fibrosis-4 index (FIB-4) categories (<1.3 low risk, 1.3–2.67 intermediate risk, >2.67 high risk) were analysed as non-invasive fibrosis risk surrogates [12,13,14].

### 2.3. Procedural Protocol

Transoesophageal echocardiography was performed within 12 h before the procedure to exclude left atrial thrombus, according to the local protocol. Ablations were performed under propofol and remifentanil-based deep sedation with preserved spontaneous ventilation. Ultrasound-guided right femoral venous access was obtained in all patients using three venous sheaths. Intravenous heparin was administered before transseptal puncture, with activated clotting time maintained at ≥300 s during left atrial instrumentation. All procedures were performed by 4 experienced electrophysiology operators using a standardised institutional PFA workflow. PFA was performed in all patients using the FARAPULSE system with a FARAWAVE pentaspline PFA catheter and dedicated generator (Boston Scientific, Marlborough, MA, USA). Catheter navigation was fluoroscopy-based and supported by rotational left atrial angiography. Pulmonary vein isolation was the procedural endpoint in all patients, and acute success was defined as entrance block in all pulmonary veins at the end of the procedure. Following ablation, fluoroscopic assessment of diaphragmatic motion was performed to evaluate phrenic nerve function. Transthoracic echocardiography was routinely performed before discharge to exclude pericardial effusion.

### 2.4. Endpoints

Procedural endpoints included total skin-to-skin procedure time, fluoroscopy time, fluoroscopy dose, number of PFA applications, application time, total heparin dose, first-pass pulmonary vein isolation, and acute pulmonary vein isolation. First-pass isolation was analysed at the patient level and defined as pulmonary vein isolation achieved after the initial planned PFA application set, without additional touch-up applications. Pulmonary vein-level first-pass isolation was not analysed because vein-level first-pass status was not systematically captured in a structured and reproducible manner across the entire cohort. Safety was assessed as a composite of any in-hospital periprocedural complication, with individual components reported descriptively.

### 2.5. Analytical Hierarchy and Statistical Analysis

The analytical hierarchy was defined according to the descriptive aims of the study. The primary analysis estimated the prevalence of HRI-detected hepatic steatosis in the analytic cohort. Secondary analyses assessed agreement between non-invasive liver-marker classifications. Procedural and safety analyses were exploratory and were performed to provide acute procedural context for the HRI-detected steatosis phenotype; they were not designed to test equivalence, non-inferiority, comparative safety, or long-term efficacy.

Continuous variables are reported as mean ± standard deviation or median [interquartile range], as appropriate, and categorical variables as number (percentage). Baseline between-group differences between patients with and without HRI-detected steatosis were described using standardised mean differences (SMD), with |SMD| > 0.3 considered a meaningful imbalance. Procedural endpoints are reported as median [interquartile range] or n (%) and were compared using the Mann–Whitney U test or Fisher’s exact test, as appropriate. For effect-size description, Hodges–Lehmann median differences (steatosis minus no steatosis) were computed for continuous procedural endpoints with bias-corrected and accelerated bootstrap 95% confidence intervals (R = 10,000). Absolute risk differences with 95% confidence intervals were reported for categorical endpoints. These effect estimates were used descriptively and may differ marginally from formal rank-based or exact test inference.

Patient-level concordance between binary liver marker classifications was assessed in available cases using Cohen’s κ, prevalence-adjusted bias-adjusted κ (PABAK), and positive and negative agreement. Concordance was interpreted primarily for the two steatosis-related markers, HRI and HSI; comparisons involving BARD and FIB-4 were considered descriptive because these scores assess fibrosis risk rather than steatosis. Correlations between continuous liver markers were assessed using Spearman’s rank correlation.

Intraobserver reproducibility of continuous HRI measurements was assessed using the intraclass correlation coefficient for absolute agreement with 95% confidence intervals. Agreement for binary HRI classification according to the primary threshold of HRI ≥ 1.28 was described using observed agreement and Cohen’s κ. Bland–Altman bias and 95% limits of agreement were reported descriptively.

For the nominal fluoroscopy dose signal, sensitivity analyses assessed robustness after adjustment for BMI and AF type, with HRI modelled as a continuous variable, and after exclusion of an influential high-dose observation. Secondary analyses were repeated using an HRI-based MASLD-compatible phenotype. No single procedural endpoint was prespecified for confirmatory testing; all procedural comparisons were interpreted as hypothesis-generating.

Given the low number of complications, safety outcomes were analysed descriptively with exact 95% confidence intervals, and no event models were fitted. This was a descriptive, exploratory study without a formal sample size calculation or adjustment for multiple comparisons; *p-values* should therefore be interpreted as hypothesis-generating. Analyses were performed in Python 3.11 (Python 3.11 (Python Software Foundation, Wilmington, DE, USA) using NumPy 1.26.4, SciPy 1.17.1, pandas 2.2.2, and statsmodels 0.14.6.

### 2.6. Ethics

The protocol was approved by the local Bioethics Committee (approval number KB/265/2023) and conducted per the Declaration of Helsinki. Written informed consent was obtained from all patients.

## 3. Results

### 3.1. Prevalence of Hepatic Steatosis and Liver Markers

**Figure 1:**
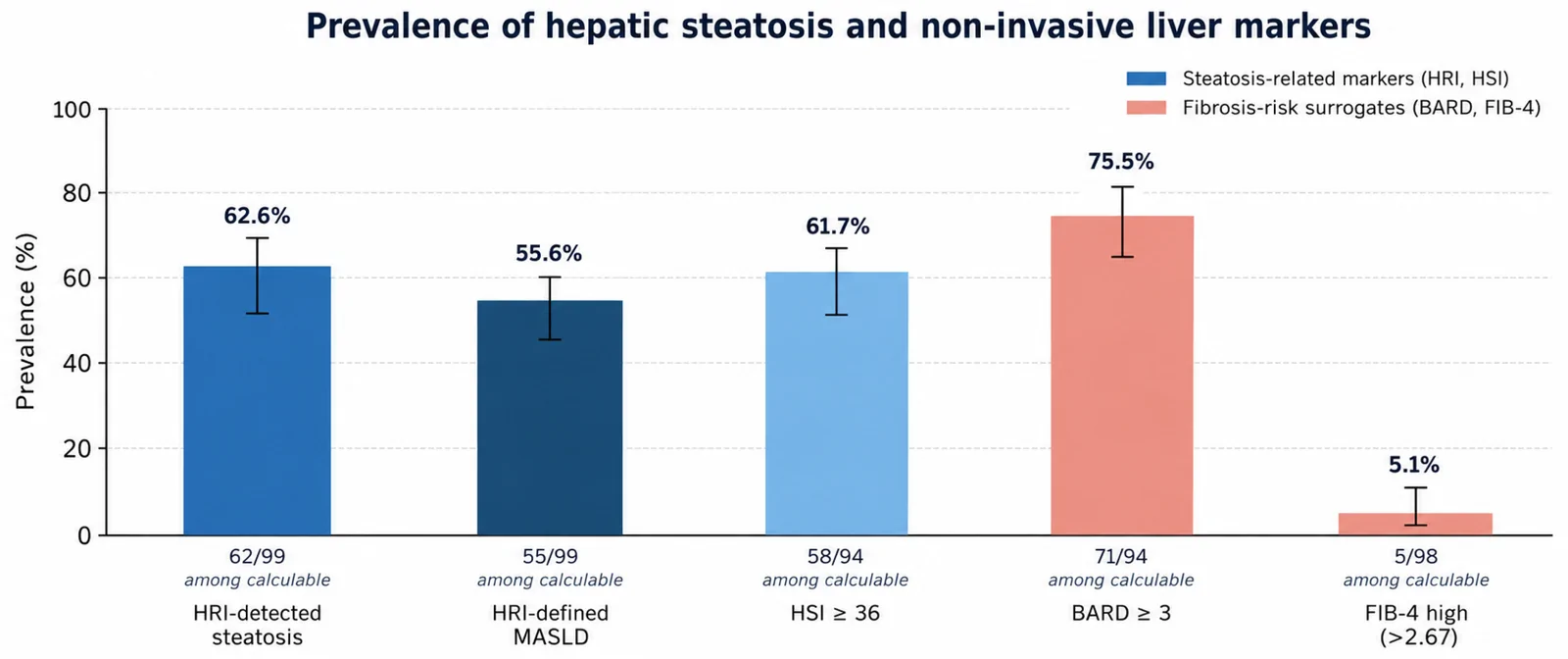
Prevalence of hepatic steatosis and non-invasive liver markers (N = 99). BARD and FIB-4 are presented as fibrosis risk surrogates and were not interpreted as steatosis markers. Bars show the proportion meeting each threshold with 95% Wilson confidence intervals; denominators are 99/99 for HRI and HRI-based MASLD-compatible phenotype and calculable cases for HSI, BARD, and FIB-4. Colour distinguishes steatosis markers (HRI, HSI) from fibrosis risk surrogates (BARD, FIB-4).

Patient selection is shown in Figure S1. The final analytic cohort included 99 patients undergoing first-time PFA with valid HRI assessment and available acute procedural data. HRI-detected hepatic steatosis was present in 62 patients (62.6%, 95% CI 52.8–71.5), corresponding to approximately two-thirds of the cohort. Continuous HRI values and prevalence estimates using alternative HRI thresholds are reported in Table S3 and Figure S4. Intraobserver reproducibility of HRI measurement was assessed in a stratified random subset of 30 patients and is reported in Table S4. Reproducibility was high, with an ICC for absolute agreement of 0.998 (95% CI 0.996–0.999), Bland–Altman bias of +0.006 (95% limits of agreement −0.033 to +0.045), and complete agreement for binary HRI ≥ 1.28 classification (30/30; Cohen’s κ = 1.00). An HRI-based MASLD-compatible phenotype was present in 55 patients (55.6%, 95% CI 45.7–65.0). Among patients with calculable secondary liver indices, the steatosis-related HSI ≥ 36 was present in 58/94 patients (61.7%). Fibrosis risk surrogates were reported separately: BARD ≥ 3 was present in 71/94 patients (75.5%), whereas high fibrosis risk by FIB-4 (>2.67) was uncommon (5/98, 5.1%), with most patients classified as low or intermediate risk (43.9% and 51.0%, respectively). This may suggest a predominantly steatosis-related phenotype without a substantial advanced fibrosis signal (Figure 1).

### 3.2. Concordance Between Liver Markers

Concordance was assessed primarily between the two steatosis-related markers, HRI and HSI. Despite similar overall prevalence, patient-level agreement was poor: observed agreement was 53%, Cohen’s κ was 0.01, and prevalence- and bias-adjusted κ was +0.06, with positive agreement of 62% and negative agreement of 39% (n = 94; Figure S2). Continuous HRI and HSI were essentially uncorrelated (Spearman ρ ≈ −0.01), whereas BARD and FIB-4 showed moderate correlation as fibrosis risk surrogates (ρ ≈ 0.51). Comparisons between steatosis-related markers and fibrosis risk surrogates were interpreted descriptively, as these indices capture different constructs. Overall, these findings indicate that non-invasive liver markers identify overlapping but non-identical patients and should not be treated as interchangeable. Because neither HRI nor HSI served as a reference standard in this study, this finding was interpreted as limited agreement between marker-based classifications rather than diagnostic discordance.

### 3.3. Baseline Characteristics

Baseline characteristics by steatosis status are shown in Table 1. The groups were broadly balanced for most variables. Patients with HRI-detected steatosis had larger waist circumference (99.7 vs. 94.7 cm; SMD +0.45) and a higher proportion of paroxysmal AF (87.1% vs. 70.3%; SMD +0.42), whereas age, sex, body mass index, hypertension, diabetes, dyslipidaemia, and platelet count were similar between groups. ALT and AST values were within the normal range in both groups.

### 3.4. Procedural Characteristics

**Figure 2:**
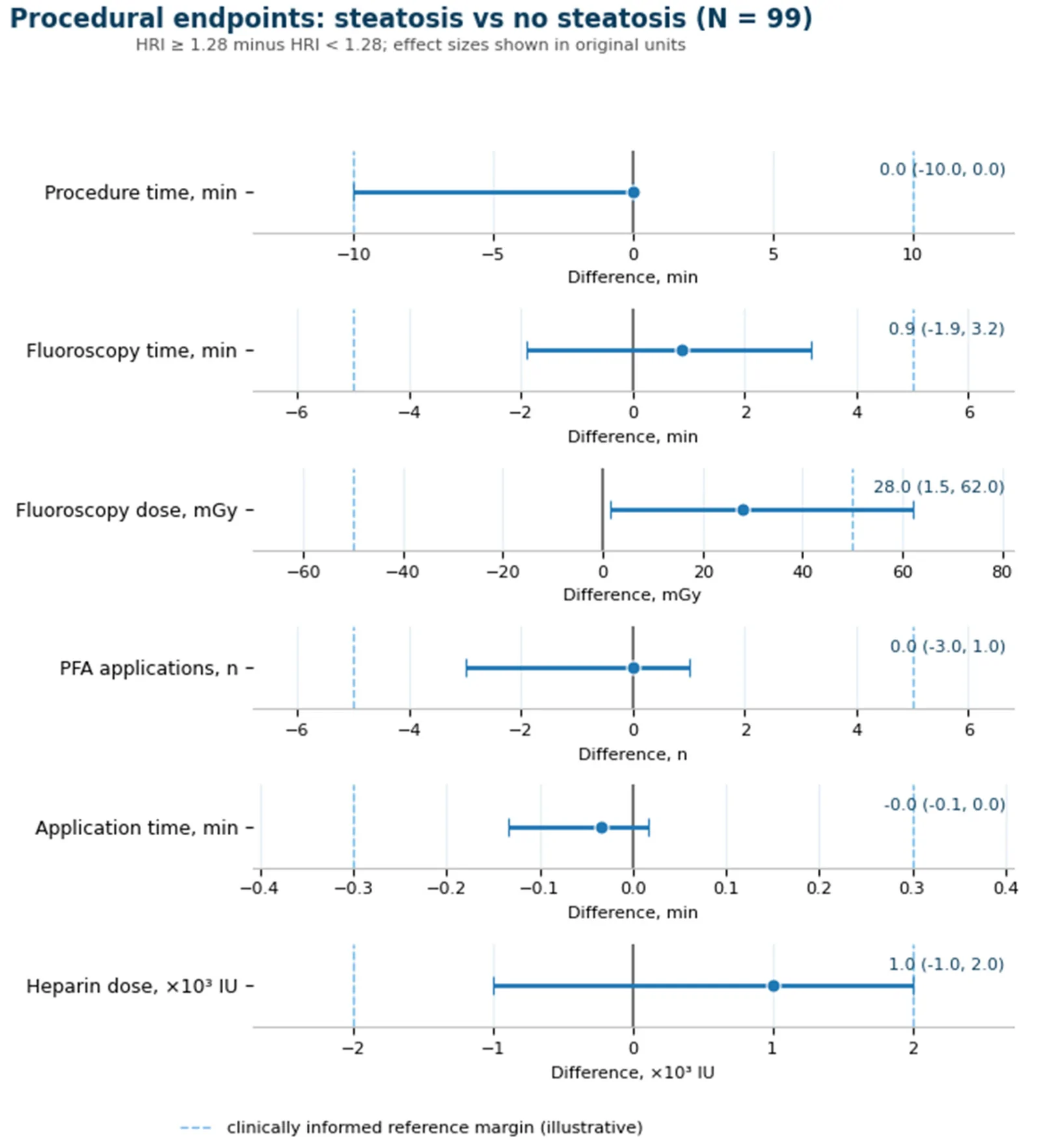
Continuous procedural metrics by hepatic steatosis status.

Procedural characteristics by steatosis status are summarised in Table 2 and Figure 2. Acute pulmonary vein isolation was achieved in all patients (99/99, 100%). First-pass isolation was high in both groups (91.9% with steatosis vs. 81.1% without steatosis; *p* = 0.124). Procedure time, fluoroscopy time, number of PFA applications, application time, and total heparin dose were broadly similar between groups. Fluoroscopy dose was modestly higher in patients with steatosis (median 226 [IQR 189–267] vs. 207 [IQR 157–241] mGy; *p* = 0.035), with a Hodges–Lehmann median difference of +28.0 mGy (95% CI +1.5 to +62.0).

Between-group differences are shown as HRI ≥ 1.28 minus HRI < 1.28. Filled circles represent Hodges–Lehmann median differences; horizontal solid lines represent bias-corrected and accelerated bootstrap 95% confidence intervals (R = 10,000); vertical dashed lines indicate clinically informed reference margins, shown for visual orientation only and not used for equivalence, non-inferiority or hypothesis testing Formal inference is reported in Table 2.

Exploratory sensitivity analyses for the nominal fluoroscopy dose difference are summarised in Table S2. The unadjusted Hodges–Lehmann median difference was +28.0 mGy (95% BCa CI +1.5 to +62.0). The estimate was attenuated after adjustment for BMI and AF type and was not supported when HRI was modelled as a continuous variable. After adjustment for waist circumference, the estimated steatosis coefficient reversed direction and was compatible with no difference (−8.2 mGy, 95% CI −25.9 to +9.4); similar results were observed after additional adjustment for AF type (−6.2 mGy, 95% CI −24.3 to +12.9). Sensitivity analyses using more conservative HRI thresholds also did not support a consistent steatosis-specific fluoroscopy dose signal. Fluoroscopy time was not increased in patients with steatosis, suggesting that the nominal dose difference, if present, more likely reflected dose rate or body habitus effects than longer screening. Accordingly, this finding was interpreted as exploratory rather than as evidence of a steatosis-specific procedural effect.

### 3.5. Periprocedural Safety

In-hospital complications were infrequent (4/99, 4.0%; 95% CI 1.1–10.0) and showed no apparent clustering by steatosis status (3/62, 4.8% vs. 1/37, 2.7%; Fisher *p* = 1.000). Events comprised one pericardial effusion not requiring intervention, one cardiac tamponade, one access site haematoma, and one intraprocedural asystole (Table 3). During the index hospitalisation, there were no strokes, transient ischaemic attacks, phrenic nerve palsies, or deaths. The study did not include structured post-discharge surveillance for late complications. Because of the low event count, safety outcomes were interpreted descriptively, and no inferential conclusions regarding comparative safety were drawn.

### 3.6. Secondary Analysis: HRI-Based MASLD-Compatible Phenotype

An HRI-based MASLD-compatible phenotype was present in 55/99 patients (55.6%) using the primary definition based on cardiometabolic criteria available across the cohort. Applying the fullest 2023 cardiometabolic criteria supported by the available data increased HRI-based MASLD-compatible phenotype prevalence to 61/99 (61.6%, 95% CI 51.8–70.6), reclassifying six additional patients. The availability of metabolic and alcohol-related variables relevant to this classification is summarised in Table S5. Because this phenotype showed high concordance with HRI-detected steatosis alone, procedural analyses by MASLD-compatible phenotype status were consistent with the primary steatosis-based analysis.

## 4. Discussion

In this prospective single-centre cohort of patients undergoing first-time PFA for AF, HRI-detected hepatic steatosis was common, affecting approximately two-thirds of patients, while the HRI-based MASLD-compatible phenotype was present in just over half. A second key finding was the poor patient-level concordance between non-invasive steatosis-related markers, indicating that marker choice materially affects phenotypic classification. Exploratorily, acute procedural characteristics were broadly similar according to steatosis status: acute pulmonary vein isolation was universal, first-pass isolation was high, and estimated differences in most procedural metrics were small or imprecise. The only nominal difference, fluoroscopy dose, was modest and inconsistent across sensitivity analyses. These data therefore characterise a common hepatic metabolic phenotype in contemporary PFA candidates, but do not establish an effect of steatosis on ablation durability or long-term rhythm outcomes.

The fluoroscopy dose comparison was one of several exploratory procedural contrasts and remains vulnerable to multiplicity and residual confounding. In particular, the signal was not supported after adjustment for waist circumference, suggesting that body habitus rather than HRI-detected steatosis itself may have contributed to the nominal unadjusted difference. Fluoroscopy dose may also be influenced by operator technique, equipment settings, the use and parameters of rotational angiography, and learning curve effects. Therefore, this finding should not be over-interpreted as evidence of a steatosis-specific procedural effect.

The high prevalence of hepatic steatosis in this cohort supports the concept that patients referred for AF ablation frequently carry an unrecognised cardiometabolic liver phenotype. In previous AF cohorts, steatotic liver disease has commonly been assessed using clinical or formula-based definitions rather than imaging-based HRI; for example, NAFLD defined by Fatty Liver Index ≥ 60 was reported in approximately 42% of patients with AF [15]. Direct comparisons are limited by differences in population selection, liver-disease nomenclature, diagnostic methods, and steatosis thresholds, but the 62.6% prevalence of HRI-detected steatosis observed in the present first-time PFA cohort appears consistent with the cardiometabolic enrichment expected among patients referred for AF ablation [9,16]. To our knowledge, HRI-based ultrasound phenotyping has not previously been systematically applied to characterise hepatic steatosis in a first-time PFA cohort. This is clinically relevant in the context of contemporary AF care, as the 2024 ESC guidelines emphasise comorbidity and risk factor management as a core component of the AF-CARE pathway [1]. Hepatic steatosis may therefore be viewed both as a manifestation of shared cardiometabolic comorbidities and as a clinically relevant comorbidity in its own right. Against this background, the present study shifts the focus from recurrence after thermal ablation to imaging-based liver metabolic phenotyping and acute procedural conduct during PFA.

The poor concordance between HRI and HSI is methodologically important. Although both are used as steatosis-related markers and showed similar overall prevalence, they classified substantially different patients. This likely reflects their different constructs: HRI is an imaging-derived liver-to-kidney echogenicity ratio, whereas HSI is a formula incorporating transaminases, body mass index, sex, and diabetes. Limited agreement between imaging-based and formula-based steatosis measures has also been described in the hepatology literature, supporting the view that this is not unique to the present AF/PFA cohort [17]. The negligible agreement after prevalence adjustment suggests that these markers were not interchangeable at the patient level in this cohort. The wider divergence between steatosis-related markers and BARD or FIB-4 was expected, as BARD and FIB-4 are fibrosis risk surrogates rather than steatosis markers. In this cohort, high FIB-4 was uncommon, supporting the interpretation of a predominantly steatosis-related phenotype without a strong advanced fibrosis signal.

Procedurally, the findings were largely reassuring but should be interpreted within the exploratory design. Acute pulmonary vein isolation was achieved in all patients, consistent with the high acute success rates reported in contemporary PFA studies. Most procedural metrics, including procedure time, fluoroscopy time, PFA applications, application time, and heparin dose, were similar between groups. Given the exploratory design and modest sample size, these observations should not be interpreted as evidence of procedural equivalence between groups. The nominally higher fluoroscopy dose in patients with steatosis was modest in absolute terms and inconsistent across sensitivity analyses. Because fluoroscopy time was not increased, the signal, if real, may reflect dose rate effects related to body habitus rather than a steatosis-specific procedural burden. This interpretation supports general radiation reduction strategies in patients with larger body size, rather than steatosis-specific procedural modification.

The lack of long-term rhythm outcome analysis is clinically important. Hepatic steatosis and MASLD may plausibly influence AF progression, atrial remodelling, and post-ablation atrial tachyarrhythmia recurrence through metabolic, inflammatory, and structural pathways. These pathways can also be interpreted within the broader framework of atrial cardiomyopathy, in which cardiometabolic conditions may contribute to structural, electrical, and functional atrial remodelling [18]. Therefore, similar acute procedural characteristics should not be interpreted as evidence that hepatic steatosis has no prognostic relevance after PFA. Rather, the present study provides imaging-based liver metabolic phenotyping and acute procedural context, while arrhythmia-free survival after PFA requires dedicated longitudinal analysis.

Three practical implications follow. First, hepatic steatosis appears common among patients referred for PFA and may identify a subgroup requiring broader cardiometabolic risk assessment beyond the index procedure. Second, the broadly similar acute procedural metrics are reassuring, but should not be interpreted as evidence of equivalent long-term efficacy or safety. Third, the limited agreement between non-invasive liver markers argues for selecting a single, mechanistically appropriate steatosis measure when designing future studies. Whether systematic pre-procedural steatosis assessment improves risk stratification or post-ablation outcomes requires prospective evaluation with rhythm follow-up.

## 5. Limitations

Several limitations should be acknowledged. First, this was a single-centre, descriptive, and exploratory study with a modest sample size and no formal sample size calculation. Several procedural endpoints were assessed, and nominal *p-values* should therefore be interpreted as hypothesis-generating rather than confirmatory. The modest fluoroscopy dose difference was inconsistent across sensitivity analyses and should not be interpreted as evidence of a steatosis-specific procedural effect. The study was not powered for equivalence, non-inferiority, or comparative safety testing.

Second, in-hospital complications were infrequent. This was expected in a contemporary PFA cohort, given the generally favourable periprocedural safety profile of this technology, but it limited the ability to compare safety outcomes between groups. Accordingly, complications were reported descriptively, with the aim of identifying any obvious safety signal or clustering of specific event types rather than establishing comparative safety.

Third, the study focused on acute procedural conduct and did not include systematic rhythm follow-up, lesion durability, or adjudicated post-blanking atrial tachyarrhythmia recurrence. This is an important limitation, because hepatic steatosis and MASLD may influence atrial remodelling, AF progression, and post-ablation rhythm outcomes despite similar acute procedural characteristics during the index procedure. Accordingly, the present findings should not be interpreted as assessing the prognostic impact of hepatic steatosis after PFA. Arrhythmia-free survival according to HRI-detected steatosis and MASLD-compatible liver metabolic phenotypes requires dedicated longitudinal evaluation.

Fourth, hepatic steatosis was assessed using ultrasound-derived HRI rather than a reference standard such as controlled attenuation parameter, MRI proton-density fat fraction, or histology. However, HRI is a non-invasive, accessible, and clinically scalable imaging-based method that can be integrated into cardiovascular workflows more readily than MRI or biopsy. Accordingly, an HRI-based MASLD-compatible phenotype should be interpreted as a pragmatic study phenotype rather than exhaustive hepatological phenotyping. All HRI measurements were performed offline by a single investigator blinded to clinical, procedural, and outcome data, using a standardised region-of-interest protocol. In response to peer review, intraobserver reproducibility was assessed in a stratified random subset and supported excellent repeatability of HRI measurements on the originally selected ultrasound frames. However, interobserver reproducibility was not assessed because the original study protocol used a single-reader design. In addition, because reproducibility assessment was performed on the originally selected frames, these results quantify reproducibility of region-of-interest placement and measurement rather than variability related to image acquisition or frame selection.

Fifth, patients with impaired renal function, absence of the right kidney, or technically inadequate liver–kidney imaging were excluded because reliable HRI assessment was not feasible. The eGFR < 60 mL/min/1.73 m^2^ criterion was applied to preserve the validity of kidney-referenced HRI measurements, as renal cortical echogenicity forms the denominator of the HRI ratio. However, this approach may have selected a somewhat healthier analytic cohort and may limit generalisability to patients with more advanced cardiometabolic or renal disease.

Sixth, the nominal fluoroscopy dose signal should be interpreted with caution. Although all procedures were performed using a single FARAPULSE platform and a broadly standardised fluoroscopy-based workflow, this comparison was one of several exploratory procedural contrasts and was therefore vulnerable to multiplicity. Fluoroscopy dose may be influenced by body habitus, waist circumference, operator technique, equipment settings, rotational angiography parameters, and learning curve effects. Additionally, sensitivity analyses adjusted for waist circumference did not support a consistent steatosis-specific fluoroscopy dose signal; however, the observational design and modest sample size limited our ability to fully account for residual confounding and between-group differences, including the baseline imbalance in AF type. In addition, pulmonary vein-level first-pass isolation could not be analysed because vein-level first-pass status was not systematically and reproducibly documented across the cohort.

Finally, the HRI-based MASLD-compatible phenotype was based on cardiometabolic data available in the study database. Core clinical cardiometabolic criteria were available in most patients and allowed identification of patients with HRI-detected steatosis and at least one documented cardiometabolic risk criterion. However, some laboratory components of the full 2023 MASLD framework, including triglycerides, HDL cholesterol, and HbA1c, were incompletely available, and alcohol exposure was not systematically quantified; therefore, MetALD and alcohol-related steatotic liver disease subtypes could not be reliably classified. This variable should therefore be interpreted as a clinically informed, MASLD-compatible study phenotype rather than exhaustive hepatological phenotyping or full SLD subclassification.

## 6. Conclusions

In this prospective first-time PFA cohort with feasible HRI assessment, HRI-detected hepatic steatosis was present in approximately two-thirds of patients and the HRI-based MASLD-compatible phenotype in just over half. HRI-detected steatosis, HSI, and fibrosis risk surrogates identified overlapping but non-identical patient groups in this cohort. Acute procedural conduct during the index hospitalisation did not reveal a consistent steatosis-specific procedural signal. These data support further study of liver metabolic phenotyping in PFA populations, but do not establish an effect of steatosis on lesion durability, long-term rhythm outcomes, or comparative safety. Dedicated prospective follow-up is required.

## Figures and Tables

**Table 1:** Baseline characteristics by hepatic steatosis (HRI ≥ 1.28).

Variable	Overall (N = 99)	Steatosis (n = 62)	No Steatosis (n = 37)	SMD
Age, years	60.5 ± 11.2	60.2 ± 11.4	61.0 ± 10.9	−0.07
Male sex	66 (66.7%)	43 (69.4%)	23 (62.2%)	+0.15
BMI, kg/m^2^	28.4 ± 4.0	28.6 ± 4.2	28.1 ± 3.7	+0.14
Waist circumference, cm	97.8 ± 11.3	99.7 ± 11.4	94.7 ± 10.7	+0.45
Paroxysmal AF	80 (80.8%)	54 (87.1%)	26 (70.3%)	+0.42
Hypertension	58 (58.6%)	37 (59.7%)	21 (56.8%)	+0.06
Diabetes mellitus	13 (13.1%)	9 (14.5%)	4 (10.8%)	+0.11
Dyslipidaemia	41 (41.4%)	24 (38.7%)	17 (45.9%)	−0.15
CHA_2_DS_2_-VASc	2.0 ± 1.5	2.1 ± 1.6	1.9 ± 1.3	+0.12
HAS-BLED	1.1 ± 0.7	1.2 ± 0.7	1.0 ± 0.6	+0.30
ALT, U/L	26.0 [22.0–34.8]	28.0 [22.0–35.0]	24.0 [20.0–33.0]	+0.08
AST, U/L	27.0 [22.0–33.0]	28.0 [23.0–33.0]	24.0 [20.0–33.0]	+0.20
Platelets, ×10^9^/L	231.7 ± 54.7	232.6 ± 54.5	230.1 ± 55.9	+0.05

Data are mean ± SD, median [IQR], or n (%). The standardised mean difference (SMD) is the primary measure of between-group difference (|SMD| > 0.3 indicating meaningful imbalance). ALT and AST are shown as median [IQR] owing to right skew. CHA_2_DS_2_-VASc and HAS-BLED were available in 76/99 and 75/99 patients, respectively; all other variables were available in ≥94/99. CHA_2_DS_2_-VASc and HAS-BLED were not required for the primary analysis and were reported descriptively in available cases only. AF, atrial fibrillation; ALT, alanine aminotransferase; AST, aspartate aminotransferase; BMI, body mass index; HRI, hepato-renal index; IQR, interquartile range; SD, standard deviation; SMD, standardised mean difference.

**Table 2:** Procedural characteristics by hepatic steatosis.

Variable	Overall (N = 99)	Steatosis (n = 62)	No Steatosis (n = 37)	Difference (95% CI) ^†^	*p* *
Procedure time, min	45.0 [40.0–57.5]	45.0 [40.0–55.0]	50.0 [40.0–60.0]	0.0 (−10.0 to 0.0)	0.535
Fluoroscopy time, min	12.7 [9.5–16.4]	12.8 [9.8–16.4]	10.8 [9.0–16.1]	+0.9 (−1.9 to +3.2)	0.479
Fluoroscopy dose, mGy	218 [177–259]	226 [189–267]	207 [157–241]	+28.0 (+1.5 to +62.0)	0.035
PFA applications, n	40 [36–43]	40 [36–44]	40 [37–43]	0.0 (−3.0 to +1.0)	0.839
Application time, min ^§^	1.7 [1.6–1.8]	1.7 [1.5–1.8]	1.7 [1.6–1.8]	0.0 (−0.1 to 0.0)	0.179
Heparin dose, ×10^3^ IU	16.0 [13.0–18.0]	16.0 [13.0–18.0]	16.0 [13.8–18.0]	+1.0 (−1.0 to +2.0)	0.446
First-pass isolation	87 (87.9%)	57 (91.9%)	30 (81.1%)	+10.9 (−2.5 to +26.8) ^‡^	0.124
Acute PV isolation	99 (100%)	62 (100%)	37 (100%)	0.0 (−9.7 to +5.9) ^‡^	1.000

Data are median [IQR] or n (%). * Mann–Whitney U for continuous endpoints and Fisher’s exact test for categorical endpoints; all continuous procedural variables were non-normally distributed. ^†^ Hodges–Lehmann median difference (steatosis − no steatosis) for continuous endpoints, with bias-corrected and accelerated bootstrap 95% confidence intervals (R = 10,000); risk difference (steatosis − no steatosis) for categorical endpoints, with Newcombe hybrid score 95% confidence intervals. ^‡^ Risk differences reported in percentage points. ^§^ Two implausible application-time values (33 and 90 min, data entry errors) were set to missing. Adjusted and sensitivity analyses for fluoroscopy dose are reported in the text. Fluoroscopy dose effect estimates were calculated in available cases. IQR, interquartile range; PFA, pulsed field ablation; PV, pulmonary vein.

**Table 3:** In-hospital periprocedural complications (descriptive).

Complication	Overall (N = 99)	Steatosis (n = 62)	No Steatosis (n = 37)
Any complication	4 (4.0%)	3 (4.8%)	1 (2.7%)
Pericardial effusion (no intervention)	1 (1.0%)	1 (1.6%)	0 (0.0%)
Cardiac tamponade	1 (1.0%)	1 (1.6%)	0 (0.0%)
Access-site haematoma	1 (1.0%)	0 (0.0%)	1 (2.7%)
Intraprocedural asystole (other)	1 (1.0%)	1 (1.6%)	0 (0.0%)
Clinically relevant bleeding	0 (0.0%)	0 (0.0%)	0 (0.0%)
Stroke/TIA	0 (0.0%)	0 (0.0%)	0 (0.0%)
Phrenic nerve palsy	0 (0.0%)	0 (0.0%)	0 (0.0%)
Death	0 (0.0%)	0 (0.0%)	0 (0.0%)

Reported descriptively owing to the low event count. No inferential testing was performed. TIA, transient ischaemic attack.

## Data Availability

The data presented in this study are available on reasonable request from the corresponding author. The data are not publicly available due to privacy and ethical restrictions.

## Supplementary Materials

The following supporting information can be downloaded at https://www.mdpi.com/article/10.3390/jcm15176805/s1, Supplementary Methods: Hepato-Renal Index: Assessment Protocol; Supplementary Results: Patient selection and marker availability; Figure S1: STROBE flow diagram of patient selection from screening to the analytic cohort; Table S1: Availability of non-invasive liver markers; Figure S2: Binary liver marker concordance; Figure S3: Spearman correlations among continuous liver markers; Table S2: Sensitivity analyses for fluoroscopy dose; Figure S4: Distribution of continuous hepato-renal index values in the analytic cohort; Table S3: Alternative-threshold sensitivity analyses for HRI-detected steatosis prevalence; Table S4: Intraobserver reproducibility of HRI measurements in a stratified random subset; Table S5: Availability of metabolic and alcohol-related variables relevant to the HRI-based MASLD-compatible phenotype. References [19,20,21,22] are cited in Supplementary File.

## Abbreviations

The following abbreviations are used in this manuscript:

AF	Atrial fibrillation
ALT	Alanine aminotransferase
AST	Aspartate aminotransferase
BARD	Body mass index, AST/ALT ratio and diabetes score
BCa	Bias-corrected and accelerated (bootstrap)
BMI	Body mass index
CI	Confidence interval
eGFR	Estimated glomerular filtration rate
EHRA	European Heart Rhythm Association
ESC	European Society of Cardiology
FIB-4	Fibrosis-4 index
HDL	High-density lipoprotein
HRI	Hepato-renal index
HSI	Hepatic Steatosis Index
ICC	Intraclass correlation coefficient
IQR	Interquartile range
MASLD	Metabolic dysfunction-associated steatotic liver disease
MRI	Magnetic resonance imaging
NAFLD	Non-alcoholic fatty liver disease
PABAK	Prevalence-adjusted bias-adjusted kappa
PFA	Pulsed field ablation
PV	Pulmonary vein
PVI	Pulmonary vein isolation
SD	Standard deviation
SMD	Standardised mean difference
TEE	Transoesophageal echocardiography
TIA	Transient ischaemic attack
