# Supplementary material for: Hepatic Steatosis in Pulsed Field Ablation for Atrial Fibrillation: Prevalence, Liver Marker Concordance and Acute Procedural Characteristics
Source: J Clin Med. 2026 Sep 2;15(17):6805. doi: 10.3390/jcm15176805 (PMC13565777; doi:10.3390/jcm15176805)
Supplement: Supplementary file 1 [file jcm-15-06805-s001.zip › jcm-4446722-supplementary.pdf]

Supplementary Materials

Supplementary Methods

Hepato-Renal Index: Assessment Protocol

Ultrasound image acquisition

Hepatic steatosis was assessed using the hepato-renal index (HRI) derived from B-mode ultrasound images. Images were acquired during routine echocardiographic ultrasound assessment using a standardised preset. Examinations were performed with patients in the supine position from the right lateral approach. Ultrasound frames included the right liver lobe and the adjacent right renal cortex within the same imaging plane whenever feasible. Gain and time-gain compensation settings were not modified during image acquisition.

Image selection and quality control

For each patient, one representative ultrasound frame was selected for HRI assessment. Images were considered eligible if homogeneous liver parenchyma and right renal cortex were simultaneously visualised and if image quality allowed reliable placement of regions of interest. Images with inadequate visualisation of the renal cortex, excessive acoustic shadowing, or relevant artefacts were considered non-diagnostic.

Region-of-interest placement

HRI measurements were performed offline using ImageJ software (National Institutes of Health, USA). Regions of interest were manually placed within homogeneous areas of the liver parenchyma and the right renal cortex, avoiding visible vessels, focal lesions and acoustic artefacts. Three regions of interest were positioned within the liver parenchyma and three within the renal cortex. Regions of interest were placed at comparable imaging depths to minimize the influence of ultrasound beam attenuation and had a fixed area of approximately 100–200 pixels^2.

HRI calculation

For each region of interest, mean grayscale values were recorded. Mean liver echogenicity was calculated as the average of the three liver region-of-interest grayscale values, and mean renal cortical echogenicity as the average of the three renal cortical region-of-interest grayscale values. HRI was calculated as the ratio of mean liver echogenicity to mean renal cortical echogenicity. Hepatic steatosis was classified using the predefined cut-off of HRI ≥1.28.

Blinding and data recording

All HRI measurements were performed offline by a single investigator blinded to clinical characteristics, procedural details and study outcomes. Raw grayscale values, averaged liver and renal echogenicity values, calculated HRI values and binary steatosis classification were recorded in a dedicated study database.

Reproducibility assessment

Intraobserver reproducibility of HRI measurement was assessed in a stratified random subset of 30 patients, including 15 patients with and 15 without HRI-detected steatosis according to the primary HRI ≥1.28 threshold. Repeat measurements were performed by the same observer using the originally selected ultrasound frames and the same region-of-interest placement protocol as in the primary analysis. During repeat measurement, the observer was blinded to the original HRI values, clinical characteristics and procedural data. Because the originally selected frames were used, this analysis assessed reproducibility of region-of-interest placement and HRI measurement rather than reproducibility of image acquisition or frame selection.

Supplementary Results

Patient selection and marker availability

Patient selection is shown in **Figure S1**. Exclusion categories were assigned hierarchically and were mutually exclusive to avoid double counting. The final analytic cohort included 99 patients undergoing first-time pulsed field ablation with valid hepato-renal index assessment and complete acute procedural data.

Availability of non-invasive liver markers is summarised in **Table S1**. HRI was available in all patients and constituted the primary steatosis measure. HSI and BARD were calculable in 94/99 patients, whereas FIB-4 was calculable in 98/99 patients. Analyses involving HSI, BARD and FIB-4 were therefore performed using available-case data.

Concordance between non-invasive liver markers

Pairwise concordance between binary liver-marker classifications is illustrated in **Figure S2**. Concordance was interpreted primarily for the two steatosis-related markers, HRI and HSI. Comparisons involving BARD and FIB-4 were considered descriptive, because these scores assess fibrosis risk rather than hepatic steatosis.

Continuous-marker correlations are shown in **Figure S3**. HRI and HSI were essentially uncorrelated, whereas BARD and FIB-4 showed moderate correlation, consistent with their shared role as fibrosis-risk surrogates.

Analyses involving HSI, BARD and FIB-4 were performed using available-case data because missingness reflected unavailable components required to calculate each index rather than outcome-dependent missingness.

Sensitivity analyses for fluoroscopy dose

Exploratory sensitivity analyses for the nominal fluoroscopy-dose difference are summarised in **Table S2**. The signal was inconsistent across modelling approaches: it was attenuated after adjustment for body habitus and AF type, was not supported when HRI was analysed as a continuous variable, and was no longer supported after adjustment for waist circumference. It was also not explained solely by the single highest-dose observation. These analyses

support interpreting the fluoroscopy-dose finding as exploratory rather than as evidence of a steatosis-specific procedural effect.

Continuous HRI distribution and alternative-threshold analyses

The distribution of continuous HRI values in the analytic cohort is shown in Figure S4. Median HRI was 1.35 [IQR 1.12–1.62] overall, 1.54 [1.40–1.83] in patients with HRI-detected steatosis according to the primary HRI ≥1.28 threshold, and 1.08 [1.03–1.17] in patients without HRI-detected steatosis. Sensitivity analyses using alternative HRI thresholds are summarised in Table S3. Alternative HRI thresholds were selected from published validation studies and used as sensitivity analyses rather than alternative diagnostic definitions. The HRI ≥1.49 threshold was selected as a higher histology-validated threshold for >5% steatosis, whereas HRI ≥1.86 was used as a threshold proposed for moderate steatosis. A more conservative specificity-oriented threshold of HRI ≥2.01 was additionally assessed.

Availability of metabolic and alcohol-related variables relevant to the HRI-based MASLD-compatible phenotype

Availability of metabolic and alcohol-related variables relevant to the pragmatic HRI-based MASLD-compatible phenotype is summarised in Table S5. Core clinical cardiometabolic criteria were largely available and allowed identification of patients with HRI-detected steatosis who had at least one documented cardiometabolic risk criterion. Laboratory components including triglycerides, HDL cholesterol and HbA1c were available only in a subset of patients and were used where available; they were not required when another cardiometabolic criterion was already documented. Alcohol questionnaire data were available in 42/99 patients; therefore, systematic quantification of alcohol exposure across the cohort was not possible, and MetALD and alcohol-related steatotic liver disease subtypes could not be reliably classified.

Figure S1. STROBE flow diagram of patient selection from screening to the analytic cohort (N = 99).

Figure S1 — STROBE flow diagram

Patient flow from screening to analytic cohort

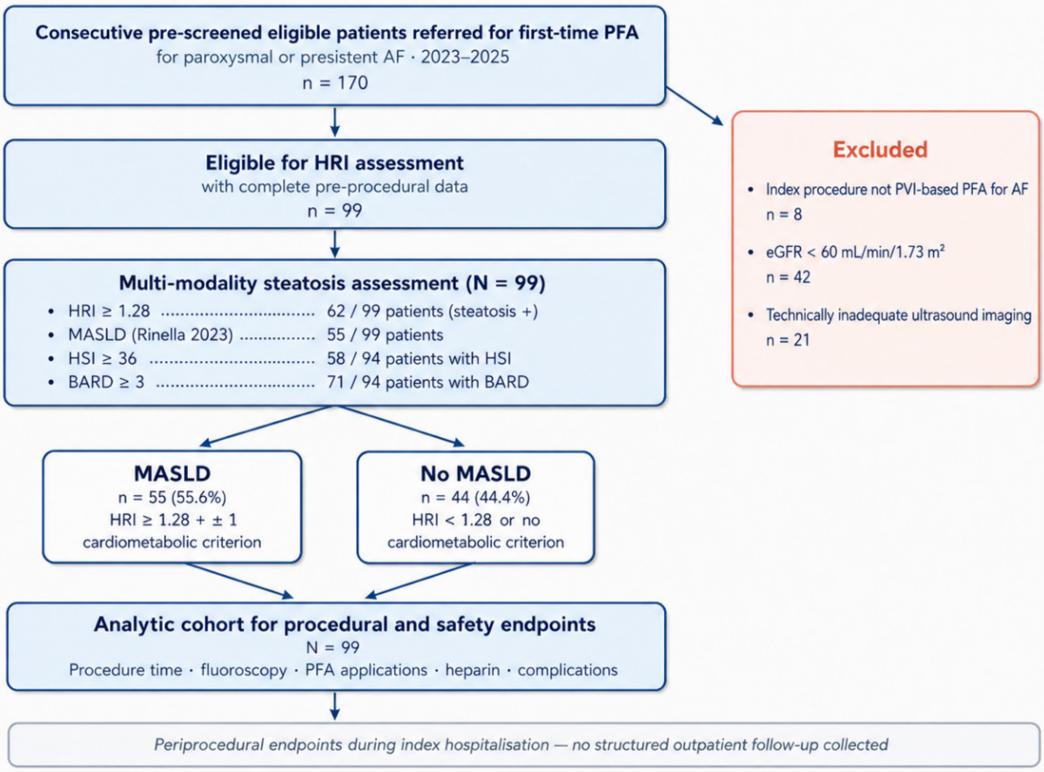

Exclusion categories were assigned hierarchically and were mutually exclusive.

Table S1. Availability of non-invasive liver markers

Marker	Available	Missing	Role
HRI	99/99	0/99	Primary steatosis marker
HSI	94/99	5/99	Secondary steatosis-related index
BARD	94/99	5/99	Fibrosis-risk surrogate
FIB-4	98/99	1/99	Fibrosis-risk surrogate

BARD, BARD score; FIB-4, Fibrosis-4 index; HRI, hepato-renal index; HSI, Hepatic Steatosis Index.

Figure S2. Binary liver-marker concordance

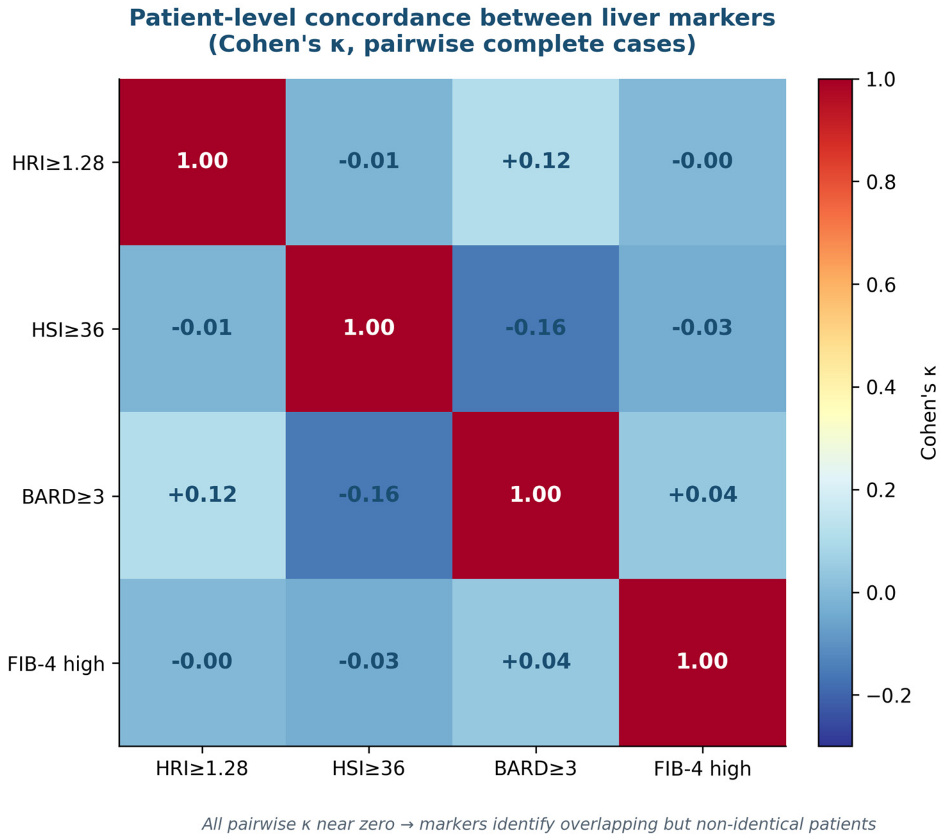

BARD, BARD score; FIB-4, Fibrosis-4 index; HRI, hepato-renal index; HSI, Hepatic Steatosis Index.

Figure S3. Spearman correlations among continuous liver markers.

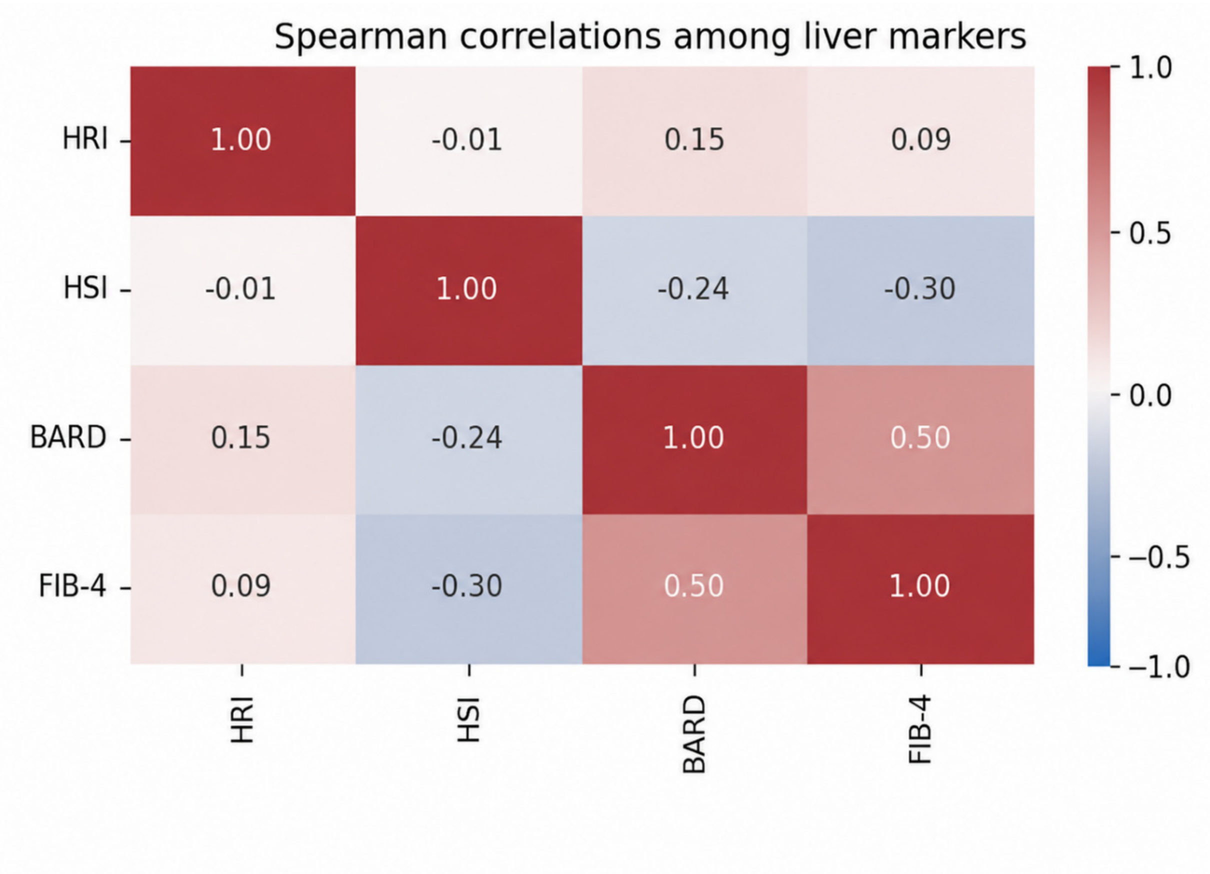

BARD, BARD score; FIB-4, Fibrosis-4 index; HRI, hepato-renal index; HSI, Hepatic Steatosis Index.

Table S2. Sensitivity analyses for fluoroscopy dose.

Analysis	n	Estimator	Effect estimate (95% CI), mGy
Unadjusted, full cohort, HRI ≥ 1.28 vs < 1.28	97	Hodges–Lehmann median difference + BCa bootstrap 95% CI	+28.0 (+1.5 to +62.0)
Adjusted for BMI	92	Quantile regression, τ = 0.50, steatosis coefficient + BCa bootstrap 95% CI	+15.0 (-7.3 to +39.8)
Adjusted for AF type, paroxysmal vs persistent	97	Quantile regression, τ = 0.50, steatosis coefficient + BCa bootstrap 95% CI	+19.0 (-25.0 to +50.6)
Adjusted for BMI + AF type	92	Quantile regression, τ = 0.50, steatosis coefficient + BCa bootstrap 95% CI	+17.2 (-5.0 to +43.6)
HRI as continuous, per 0.1-unit increase	97	Quantile regression, τ = 0.50, HRI coefficient per 0.1-unit increase + BCa bootstrap 95% CI	+0.4 (-3.9 to +5.4)
Excluding the single highest fluoroscopy-dose observation	98	Hodges–Lehmann median difference + BCa bootstrap 95% CI	+26.0 (+1.0 to +58.0)
Using HSI ≥ 36 definition instead of HRI ≥ 1.28	92	Hodges–Lehmann median difference + BCa bootstrap 95% CI	+27.0 (-2.0 to +52.0)
Using HRI-based MASLD-compatible phenotype instead of HRI-detected steatosis	97	Hodges–Lehmann median difference + BCa bootstrap 95% CI	+25.0 (+0.0 to +54.0)
Adjusted for waist circumference	84	Quantile regression, τ = 0.50, steatosis coefficient + BCa bootstrap 95% CI	-8.2 (-25.9 to +9.4)
Adjusted for waist circumference + AF type	84	Quantile regression, τ = 0.50, steatosis coefficient + BCa bootstrap 95% CI	-6.2 (-24.3 to +12.9)
Using HRI ≥ 1.49 definition instead of ≥ 1.28	97	Hodges–Lehmann median difference + BCa bootstrap 95% CI	+8.0 (-18.0 to +35.0)
Using HRI ≥ 1.86 definition instead of ≥ 1.28	97	Hodges–Lehmann median difference + BCa bootstrap 95% CI	-2.0 (-41.0 to +33.0)
Using HRI ≥ 2.01 definition instead of ≥ 1.28	97	Hodges–Lehmann median difference + BCa bootstrap 95% CI	+2.0 (-47.0 to +59.0)

The signal was inconsistent across modelling approaches: it was attenuated after adjustment for BMI and AF type, was not supported when HRI was analysed as a continuous variable, and was no longer supported after adjustment for waist circumference. It was also not explained solely by the single highest-dose observation. These analyses support interpreting the fluoroscopy-dose finding as exploratory rather than as evidence of a steatosis-specific procedural effect.

All effects are between-group differences in fluoroscopy dose. Positive values indicate higher fluoroscopy dose in patients with the exposure phenotype. The reference category for HRI-detected steatosis was HRI <1.28; for AF type, persistent AF. P values are not reported because these analyses were descriptive and exploratory; formal inference for the unadjusted HRI-based contrast is reported in Table 2.

AF, atrial fibrillation; BCa, bias-corrected and accelerated; BMI, body mass index; CI, confidence interval; HRI, hepato-renal index; HSI, Hepatic Steatosis Index; MASLD, metabolic dysfunction-associated steatotic liver disease.

Figure S4. Distribution of continuous hepato-renal index values in the analytic cohort.

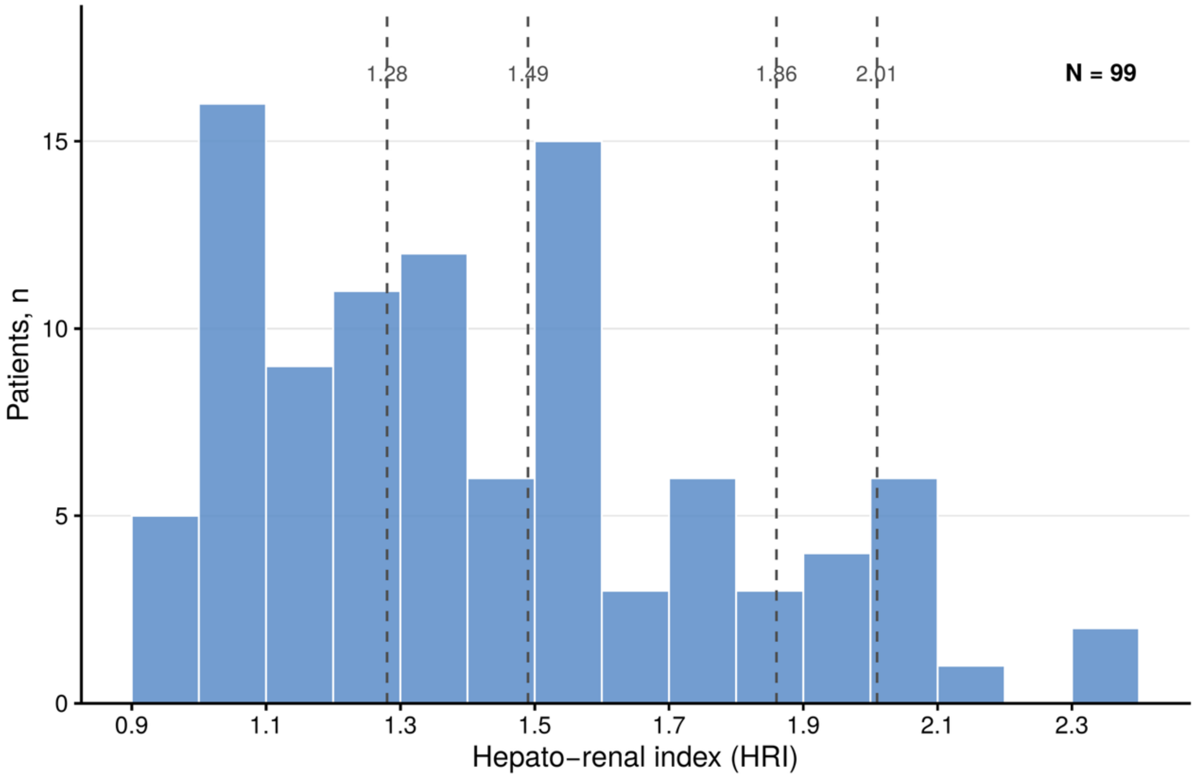

The figure shows the distribution of HRI values among patients undergoing first-time PFA with valid HRI assessment. The primary threshold for HRI-detected hepatic steatosis was HRI ≥1.28. Additional vertical reference lines indicate alternative HRI thresholds used in sensitivity analyses, where applicable.

Table S3. Alternative-threshold sensitivity analyses for HRI-detected steatosis prevalence.

Threshold	n/N (%)	95% CI	Agreement with HRI ≥ 1.28, κ

HRI ≥ 1.28 (primary definition)	62/99 (62.6%)	52.8–71.5	—
HRI ≥ 1.49 (Webb et al.)	43/99 (43.4%)	34.1–53.3	0.63
HRI ≥ 1.86 (moderate steatosis, ≥25%)	15/99 (15.2%)	9.4–23.5	—
HRI ≥ 2.01 (Chauhan et al.)	8/99 (8.1%)	4.2–15.1	0.10

CI, confidence interval (Wilson); HRI, hepato-renal index; κ, Cohen's kappa versus the primary HRI ≥ 1.28 classification. Median [IQR] HRI values are reported in the text above. Thresholds were selected from published validation studies and used for sensitivity analyses only. The primary HRI ≥1.28 threshold was based on Marshall et al. [10]. Alternative thresholds included HRI ≥1.49 and ≥1.86 from Webb et al. [S1], and HRI ≥2.01 from Chauhan et al. [S3]. Closely similar sensitivity-oriented thresholds have also been reported against 3T proton magnetic resonance spectroscopy [S4]. Published HRI cut-offs vary across validation cohorts, reference standards and acquisition protocols, as summarised in a recent systematic review and meta-analysis [S2]. These thresholds were not interpreted as competing diagnostic definitions

Supplementary References:

S1. Webb M, Yeshua H, Zelber-Sagi S, Santo E, Brazowski E, Halpern Z, et al. Diagnostic value of a computerized hepatorenal index for sonographic quantification of liver steatosis. AJR Am J Roentgenol. 2009;192(4):909-14. doi:10.2214/AJR.07.4016.

S2. Wang JL, Dong J, Wang SR, Cao CL, Tong J, Li W, et al. Reliability of ultrasonic hepatorenal indices in assessing hepatic steatosis: a systematic review and meta-analysis. J Clin Ultrasound. 2026;54(1):164-76. doi:10.1002/jcu.70006.

S3. Chauhan A, Sultan LR, Furth EE, Jones LP, Khungar V, Sehgal CM. Diagnostic accuracy of hepatorenal index in the detection and grading of hepatic steatosis. J Clin Ultrasound. 2016;44(9):580-6.

S4. Martín-Rodríguez JL, Arrebola JP, Jiménez-Moleón JJ, Olea N, González-Calvin JL. Sonographic quantification of a hepato-renal index for the assessment of hepatic steatosis in comparison with 3T proton magnetic resonance spectroscopy. Eur J Gastroenterol Hepatol. 2014;26(1):88-94.

Table S4. Intraobserver reproducibility of HRI measurements in a stratified random subset.

Comparison	n	ICC (95% CI)	Bland–Altman bias (95% LoA)
Intraobserver (original vs repeat measurement)	30	0.998 (95% CI 0.996–0.999)	+0.006 (−0.033 to +0.045)
Binary classification (HRI ≥ 1.28), Cohen's κ	30	κ = 1.00	—

ICC, intraclass correlation coefficient (two-way random effects, absolute agreement, single measurement); LoA, limits of agreement; HRI, hepato-renal index. Bland–Altman bias is expressed as the repeat measurement minus the original measurement. The subset

comprised 15 patients with and 15 without HRI-detected steatosis (HRI range 0.96–2.08); re-reading used the originally selected ultrasound frames, and binary classification agreement was complete (30/30).

Table S5. Availability of metabolic and alcohol-related variables relevant to the HRI-based MASLD-compatible phenotype (N = 99).

Criterion	Available, n/99 (%)	Missing, n (%)	Component detail
Body-mass index	94/99 (94.9%)	5 (5.1%)	—
Waist circumference	85/99 (85.9%)	14 (14.1%)	—
Elevated blood pressure or treated hypertension	99/99 (100%)	0 (0%)	diagnosis or antihypertensive treatment
Diabetes or dysglycaemia	99/99 (100%)	0 (0%)	diagnosis, HbA1c or fasting glucose
Triglycerides	24/99 (24.2%)	75 (75.8%)	—
HDL cholesterol	24/99 (24.2%)	75 (75.8%)	—
HbA1c	19/99 (19.2%)	80 (80.8%)	—
Alcohol exposure	42/99 (42.4%)	57 (57.6%)	AUDIT/CAGE, where recorded

Core clinical cardiometabolic criteria were largely available and were sufficient to identify patients with HRI-detected steatosis who had at least one documented cardiometabolic risk criterion. Serum triglycerides, HDL cholesterol and HbA1c were available only in a subset of patients; therefore, these laboratory variables were used where available but were not required when other cardiometabolic criteria were already documented. Alcohol questionnaires (AUDIT or CAGE) were recorded in 42/99 patients; systematic quantification of alcohol exposure across the cohort was therefore not possible, and MetALD and alcohol-related steatotic liver disease subtypes were not classifiable. HDL, high-density lipoprotein; HbA1c, glycated haemoglobin.
